# Polycyclic aromatic hydrocarbons (PAHs) in soils of an industrial area in semi-arid Uzbekistan: spatial distribution, relationship with trace metals and risk assessment

**DOI:** 10.1007/s10653-021-00974-3

**Published:** 2021-05-26

**Authors:** Benjamin A. Musa Bandowe, Nosir Shukurov, Sophia Leimer, Michael Kersten, Yosef Steinberger, Wolfgang Wilcke

**Affiliations:** 1 Multiphase Chemistry Department, Max-Planck Institute for Chemistry, Hahn-Meitner-Weg 1, 55128 Mainz, Germany; 2 Institute of Geology and Geophysics, State Committee of the Republic of Uzbekistan for Geology and Mineral Resources, Olimlar street 64, Tashkent, Uzbekistan 100041; 3 Geosciences Institute, Johannes Gutenberg-University, 55099 Mainz, Germany; 4 Institute of Geography and Geoecology, Karlsruhe Institute of Technology (KIT), Reinhard-Baumeister-Platz 1, 76131 Karlsruhe, Germany; 5 The Mina and Everard Goodman, Faculty of Life Sciences, Bar-Ilan University, 52900 Ramat-Gan, Israel

**Keywords:** Pollution transect, Metal mining, Smelting area, Soil health, Central Asia

## Abstract

The concentrations, composition patterns, transport and fate of PAHs in semi-arid and arid soils such as in Central Asia are not well known. Such knowledge is required to manage the risk posed by these toxic chemicals to humans and ecosystems in these regions. To fill this knowledge gap, we determined the concentrations of 21 parent PAHs, 4,5-methylenephenanthrene, 6 alkylated PAHs, and biphenyl in soils from 11 sampling locations (0–10, 10–20 cm soil depths) along a 20-km transect downwind from the Almalyk metal mining and metallurgical industrial complex (Almalyk MMC), Uzbekistan. The concentrations of Σ29 PAHs and Σ16 US-EPA PAHs were 41–2670 ng g^−1^ and 29–1940 ng g^−1^, respectively. The highest concentration of Σ29 PAHs occurred in the immediate vicinity of the copper smelting factory of the Almalyk MMC. The concentrations in topsoil decreased substantially to a value of ≤ 200 ng g^−1^ (considered as background concentration) at ≥ 2 km away from the factory. Low molecular weight PAHs dominated the PAH mixtures at less contaminated sites and high molecular weight PAHs at the most contaminated site. The concentration of Σ16 US-EPA PAHs did not exceed the precautionary values set by the soil quality guidelines of, e.g., Switzerland and Germany. Similarly, the benzo[*a*]pyrene equivalent concentration in soils near the Almalyk MMC did not exceed the value set by the Canadian guidelines for the protection of humans from carcinogenic PAHs in soils. Consequently, the cancer risk due to exposure to PAHs in these soils can be considered as low.

**Supplementary Information:**

The online version contains supplementary material available at 10.1007/s10653-021-00974-3.

## Introduction

Polycyclic aromatic hydrocarbons (PAHs) are formed and emitted into the environment from combustion of fossil fuels and biomass in the industry, power plants, gas production sites, traffic/transport (vehicles, ships etc.), household activities (e.g., cooking, heating), municipal activities (e.g., waste incineration) as well as burning of agricultural waste and vegetation fires (Lima et al., 2005; Wilcke, 2000; Wild and Jones, 1995). PAHs also occur in liquid and solid fossil fuels such as coal, crude oil and refined petroleum products and can therefore also be emitted during their exploration/mining, refining, distribution and storage (Achten and Hofmann, 2009; Neff et al., 2005). PAHs that are emitted into the atmosphere can undergo long-range transport to distant and pristine ecosystems (such as high mountain areas, the Arctic and Antarctic) that are far from the point sources (Ashayeri et al., 2018; De Laender et al., 2011; Keyte et al., 2013; Li et al., 2020). Besides long-range transport, there are also biological sources that can contribute to the PAH concentrations in background soils, albeit at low concentrations (Wilcke, 2007). The largest proportion of all the PAHs released into the environment is ultimately deposited in soils (e.g., 90% in the UK, Wild and Jones, 1995). The composition of the PAH mixtures in the environment depends on their sources, because the combustion of different materials under different conditions produces different PAH mixtures (Lima et al., 2005; Tobiszewski and Namiesnik, 2012).

Soils contaminated with PAHs can pose a health risk to humans, livestock and wildlife, as well as an ecotoxicological risk to the soil biome, soil functions, plants, the quality of air and aquatic life (CCME, 2010; Douben, 2003; IARC, 2010). The transport and environmental fate of PAHs depends on their chemical properties and environmental conditions (CCME, 2010; Neff et al., 2005; Wilcke, 2000). Post-emission transport, transformation and other processes which might modify the original composition of PAH mixtures are controlled by compound properties, soil properties, vegetation and other ambient conditions (Katsoyiannis et al., 2011; Keyte et al., 2013; Tobiszewski and Namiesnik, 2012; Wilcke et al., 2014b). Such transformation processes have implications for source identification, concentrations, spatial distribution and impact.

The characterization of the PAHs in soils and their associated risk has been a major research activity in Europe, North America and China (Arp et al., 2014; Bandowe et al., 2019b; Cai et al., 2008; CCME, 2010; Davie-Martin et al., 2017; Desaules et al., 2008; Sun et al., 2018; Wilcke, 2007). In several countries, regulatory limits and procedures are defined to assess risks to human and ecosystem health, and to identify soils that need to be remediated (Desaules et al., 2008; CCME, 2010).

To the best of our knowledge, only three published studies have characterized the PAH burden of soils in the Central Asian regions (Bandowe et al., 2010; Li et al., 2020; Zhao et al., 2017). Consequently, little is known about how emissions from different sources in combination with climatic/meteorological, land-use and soil biogeochemical properties affect the PAH concentrations in soils of central Asian eco-regions with their frequently semi-arid to arid conditions. Semi-arid to arid conditions imply limited leaching because of low rainfall, but possibly a strong effect of photochemical reactions because of long and intense sunshine durations. On the other hand, steppe soils are known for their intense biological activity resulting in a strong accumulation of organic matter during the short humid spring season (Driessen and Deckers, 2001), which might favor PAH bioturbation and storage in soil, but possibly also PAH degradation. Several clusters of heavy industries including coal mining, metal mining and smelting, refining of oil/gas and chemical industries were or are still operating in resource-rich areas in Central Asia (Sharov et al., 2016). The negative impact of organic pollutants emitted from such industries on the environmental quality and human health in Central Asia are not well characterized (Muntean et al., 2003; Sharov et al., 2016).

One such major industrial site is the Almalyk metal mining and metallurgical industrial complex (MMC), located near Tashkent, the capital of Uzbekistan. Large quantities of air pollutants have been released from this industrial complex in the past. Typical air pollutants (SO_2_, NO_*x*_, black carbon) released from this industrial complex represent about 13% of all air pollutants emitted in Uzbekistan (UNECE, 2001a, b). The local ecosystem is visibly impacted by emissions and waste from this industrial site (Kodirov and Shukurov, 2009). Previous reconnaissance studies in this region have characterized the depth and spatial distribution of trace metal contaminants in the soil and their impact on soil health (Pen-Mouratov et al., 2008; Shukurov et al., 2014). It has been found that there is an inverse relationship between the concentrations of several trace metals and soil biological properties like the health of the nematode population (Pen-Mouratov et al., 2008; Shukurov et al., 2005, 2014). Our hypothesis was that the operations of the Almalyk MMC could also release PAHs into the soil. However, the levels, spatial distribution and composition of the PAH mixtures have not yet been characterized. The risk eventually associated with anthropogenic PAH mixtures in soils therefore remains unknown.

We aimed to conduct a case study of PAHs in soils near a heavy-industrial complex in semi-arid Central Asia. The specific aims of this study were (a) to determine the spatial variation of the concentration and composition of PAH mixtures in soils along a 20-km transect downwind of the Almalyk MMC, (b) to assess if vertical translocation of PAHs in soil is dominated by leaching or bioturbation, (c) to evaluate relationships between soil properties (pH, organic C concentrations), trace metal concentrations (taken from a previous study), and PAH compositions/concentrations, and (d) to conduct a risk assessment to determine the possible negative impact of the PAHs to human and soil health.

## Materials and methods

### Description of the study site

**Fig. 1:**
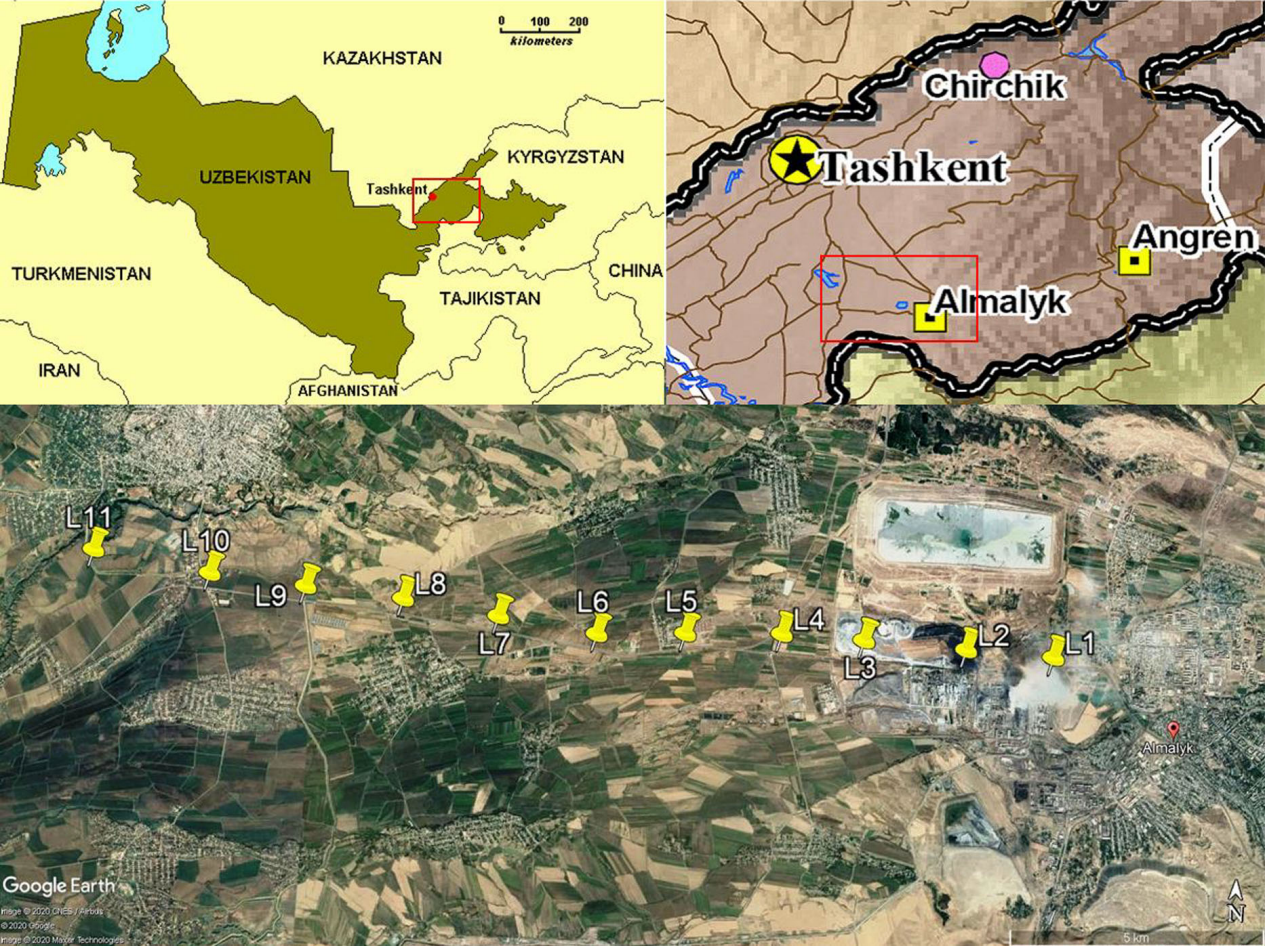
Location of the study area (aerial photograph is from Google Earth) with sampling sites L1–L11

The study site is situated at 65 km distance from Tashkent along the Akhangaran river valley (on the right-side tributary of the Sir-Darya river), which lies at the foothills between the Chatkal and Kurama mountain ranges (Western Tien-Shan), extending into the southeast part of the Tashkent region of the Republic of Uzbekistan (Fig. 1).

The city of Almalyk was founded in 1951 and integrated several settlements with inhabitants exploiting the rich mineral resources of the Kurama Mountains. The city has become an important center of nonferrous metallurgy and the most important industrial center of Uzbekistan (Encyclopaedia Britanica, 2018). The Almalyk MMC is one of the largest industrial sites in Uzbekistan and it includes a metal mining and processing enterprise, as well as metallurgical and chemical plants located in the flat bottom of the Akhangaran river valley near the city of Almalyk (40° 50′ N–69° 34′ E). Almalyk MMC produces refined copper, gold, silver, lead, metallic zinc and other products. It has the capacity to process about 25 million tons of ore per year, with an annual metal-producing capacity of 101,000 tons of Cu, 70,000 tons of Zn and 80,000 tons of Pb (Safirova, 2019). Because of a lack of efficient exhaust-treatment facilities, the Almalyk MMC is a major source of air pollution in Uzbekistan. According to an environmental report from 2000, the Almalyk MMC was found to emit about 100,000 tons of toxic substances (SO_2_, NO_X_, sulfuric acid, black carbon, heavy metals, arsenic, etc.) per year (Shukurov et al., 2005, 2014; UNECE, 2001a, b).

The research area is a mountain valley with a large variability of seasonal and daily air temperatures and wind directions. Thermal inversions cause cyclic circulation of air masses and pendulum distributions of dust and gas-smoke emissions from the industrial enterprises (Shukurov et al., 2005, 2014). The prevalent wind directions in the study area are westerly and southwesterly. Moreover, the study area is surrounded by a mountain chain, which creates poor conditions for air circulation. This further worsens the air pollution and the secondary pollution of soils and vegetation in the valley. Since 1994, the State Committee for Geology and Mineral Resources has environmentally monitored the Almalyk MMC. The results showed that groundwater, soil and vegetation are highly contaminated with trace metals such as lead, copper, zinc, selenium, tungsten, cobalt, cadmium and arsenic (UNECE, 2010).

The study area has a continental climate with hot and dry summers and short, cold winters. The coldest winter month is January with a mean temperature of 2.8 °C, but the temperature occasionally drops down to − 38 °C. The hottest summer months are July and August with mean temperatures in the foothills of 25–30 °C. A summer temperature of 42–47 °C is common with occasional peaks > 50 °C. Mean annual rainfall is 100–200 mm, which is lower than mean annual evaporation (UNECE, 2001a, b). In July and August, almost no rainfall occurs. The vegetation cover in the study area is dominated by annual and perennial plants. The most common genera include *Astragalus*, *Stipa*, *Medicago* and *Artemisia*. The soils at the study area belong to the Lithosols (IUSS Working Group WRB, 2014), shallow, stone-rich soils with high concentrations of CaCO_3_ buffering the pH near neutral values, therefore contributing to immobilization and accumulation of metals in the topsoil (Shukurov et al., 2014).

### Soil sampling and characterization

Soil samples were collected in June 2005 along the 20 km E–W Akhangaran river valley transect downwind from the Almalyk MMC (Fig. 1; Table 1).

**Table 1:** Sampling locations, land use and chemical properties of soils from 11 sampling locations along the 20 km transect away from the Almalyk industrial complex

Sampling locations and distance to industrial complex	Sampling depth (cm)	GPC coordinates	Land use	pH	*C*_org_ (%)
*L*_1_—0 km	0–10	40°51′40″N/69°33′18″E	Grassland at the border of Cu processing factory	7.9	0.5
	10–20			7.9	0.5
*L*_2_—2 km	0–10	40°51′41″N/69°32′4″E	Grassland nearby the Cu smelter slag wastes and a chemical factory	7.7	0.8
	10–20			7.7	0.7
*L*_3_—4 km	0–10	40°51′45″N/69°30′41″E	Grassland nearby the phosphogypsum landfills of a chemical factory	7.8	0.6
	10–20			7.8	0.5
*L*_4_—6 km	0–10	40°51′47″N/69°29′21″E	Grassland along the Almalyk-Buka main road	7.8	0.7
	10–20			8.1	0.6
*L*_5_—8 km	0–10	40°51′43″N/69°28′10″E	Grassland in agricultural area along the Almalyk-Buka main road	7.9	0.5
	10–20			7.9	0.5
*L*_6_—10 km	0–10	40°51′42″N/69°26′39″E	Grassland in agricultural area along the Almalyk-Buka main road	7.9	1.2
	10–20			8.1	1.1
*L*_7_—12 km	0–10	40°51′52″N/69°25′23″E	Grassland in agricultural area along the Almalyk-Buka main road	7.9	0.9
	10–20			8.1	0.6
*L*_8_—14 km	0–10	40°51′58″N/69°24′45″E	Grassland in agricultural area along the Almalyk-Buka main road	8.1	0.9
	10–20			8.0	0.7
*L*_9_—16 km	0–10	40°52′2″N/69°22′37″E	Grassland in agricultural area along the Almalyk-Buka main road	8.1	0.6
	10–20			8.2	0.5
*L*_10_—18 km	0–10	40°52′10″N/69°21′18″E	Grassland in agricultural area along the Almalyk-Buka main road	8.2	0.8
	10–20			8.1	0.6
*L*_11_—20 km	0–10	40°52′61″N/69°19′52″E	Grassland in agricultural area along the Almalyk-Buka main road	8.2	0.8
	10–20			8.1	0.6

Soils were sampled at 11 locations with 2 km distance to each other beginning from the Almalyk copper refinery and smelting factory and ending near Pskent town, crossing the Almalyk mineral fertilizers factory, industrial landfills (phosphogypsum, metallurgical slags) and agricultural lands. At each sampling point, mineral soil samples were collected from two depths layers (0–10 and 10–20 cm). Three random replicate samples at a minimum distance of 2 m from each other were collected at each location from both soil layers and transported to the laboratory. The soils were air-dried, sieved (< 2 mm) and kept refrigerated at 4 °C until chemical analysis.

Residual soil moisture of the field fresh soils was measured gravimetrically by drying the subsamples to a constant weight at 105 °C. Soil pH was determined in a suspension of a subsample in deionized water (soil:solution ratio 1:2) with a potentiometric glass electrode. The organic carbon (*C*_org_) concentration was determined using a modification of the method of Rowell (1994) based on organic matter oxidation by potassium dichromate.

### Extraction and analysis of PAH concentrations

The concentrations of PAHs in all samples [Almalyk soils, certified reference soil material ERM-CC013a (BAM, Berlin, Germany), and blanks] were analyzed by the methods described previously (Bandowe and Wilcke, 2010; Bandowe et al., 2010, 2011). In brief, about 15 g of each Almalyk soil subsample, or ca. 0.2 g of certified reference material, were weighed and mixed with inert bulk material (Isolute HM-N, Biotage, Uppsala, Sweden). Each mixture was transferred into a separate 33-mL accelerated solvent extractor (ASE) cell and spiked with 50 µL of a mixture of 7 deuterated PAHs (naphthalene-D_8_, acenaphthene-D_10_, phenanthrene-D_10_, pyrene-D_10_, chrysene-D_12_, perylene-D_12_ and benzo[*g*,*h*,*i*]perylene-D_12_) each with a concentration of 10 μg mL^−1^ in toluene. Each sample (soils and procedural blanks) was extracted by pressurized liquid extraction using an ASE 200 (Dionex, Sunnyvale, CA, USA). Each sample was extracted twice, first with dichloromethane (CH_2_Cl_2_), followed by a second extraction with CH_3_COCH_3_/CH_2_Cl_2_/CF_3_COOH (1%) [250:125:1 v/v/v]. The two extracts of each sample were combined, 10 mL hexane were added and rotary evaporated (at 35 °C) to < 1 mL. The concentrated extracts were transferred onto a silica gel column (3 g, 10% deactivated) for cleaning up/fractionation. The target compounds were eluted from the column with 15 mL hexane/dichloromethane (5:1 v/v). The eluate was spiked with three drops of toluene, rotary evaporated to about 0.5 mL and transferred to a 1.5-mL vial for gas chromatographic/mass spectrometric (GC/MS) measurement. The target compounds in our extracts were separated, identified and quantified using a gas chromatograph (Agilent 7890 A GC) coupled to a mass selective detector (Agilent 5975 MSD), operating in the electron impact ionization mode with selected ion monitoring. All data recording and processing were done with the Agilent MSD Chem Station software package. Target compounds were quantified by the internal standard technique as described by Kjeller (1998) using ten calibration standards prepared from target compound standards each spiked with a constant concentration of the internal standard mixture (i.e., 7 deuterated PAHs).

### Analytical quality control and assurance

All organic solvents used in the analysis were of high purity pesticide grade obtained from Carlo Erba (Milan, Italy). All labware was washed with soap in a dishwasher and dried in an oven. Non-volumetric glassware and metallic parts were baked in an oven at 300 °C for 24 h. Glassware was further rinsed with high-purity acetone immediately before use. We analyzed several blanks (made of inert bulk material, Isolute HM-N). The mass of a target compound detected in the blanks was deducted from that in our samples to correct for contamination during sample preparation. Some samples were extracted and analyzed in replicate to check that the analysis ran reproducibly. The relative standard deviation (RSD) of the Σ29 PAH concentrations ranged from 1.2 to 6.7% in our replicate measurements. The performance of the analytical method has previously been thoroughly evaluated and validated (Bandowe and Wilcke, 2010; Lundstedt et al., 2014). We also continuously monitor the method through analysis of certified reference soil material (ERM-CC013a, Table S1).

### Calculations and statistical evaluation

The sum of the concentrations of all target compounds, of all parent-PAHs and of the 16 US-EPA priority PAHs are referred to as ∑29 PAHs, ∑21 PAHs and ∑US-EPA PAHs, respectively. The sum of the concentrations of parent-PAHs with 2–3 rings (low-molecular weight PAHs) and with 4–6 rings (high-molecular weight PAHs) are referred to as ∑LMW-PAHs and ∑HMW-PAHs, respectively. The octanol–water partition coefficient (K_OW_) of the individual PAHs was estimated with the K_OW_WIN version 1.67 EPI Suite™ of the U.S. Environmental Protection Agency (EPA) (http://www.epa.gov/opptintr/exposure/pubs/episuitedl.htm).

We calculated the Pearson correlation between the available variables using the R statistical software (R Core Team, 2019). Variables with more than three zeros, or more than three missing values (out of 11 values), were excluded from the analysis (Table S2, Supporting Information). Normal distribution of the variables was tested with the Lilliefors normality test using the R function *lillie.test()* from the R package *nortest* (Gross and Ligges, 2015). Prior to the correlation analysis, some variables had to be log-transformed to approximate normal distribution as shown in Table S2. For a few variables, which are marked with an asterisk in Table S2, only an approximate normal distribution was reached. Statistical significance was set at *p* < 0.05.

The PAH concentrations in soils were evaluated for their possible negative impact on human and environmental health based on a risk assessment approach by Canadian regulations (CCME, 2010). The risk of carcinogenic impact on humans through direct contact to soils contaminated with PAHs was evaluated by comparing calculated benzo[*a*]pyrene Total Potency Equivalents (B[*a*]P TPE = ΣC_PAHi_*PEF_i_), where the C_PAHi_ are the concentrations of carcinogenic PAHs in soil, and PEF_*i*_ are their potency equivalency factors (CCME, 2010).

## Results and discussion

### Concentrations, spatial distribution and composition of the mixtures of PAHs

**Fig. 2:**
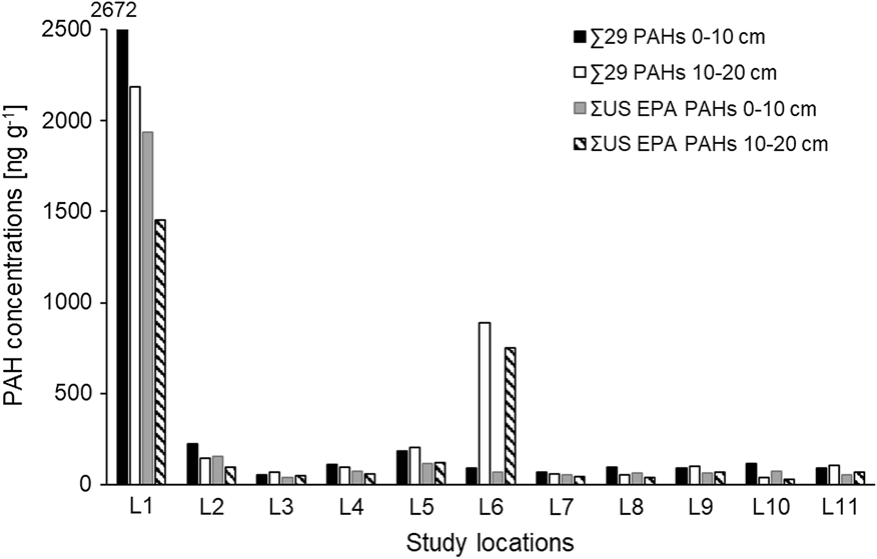
Concentration (ng g^−1^) of ∑29 PAHs and ΣUS-EPA PAHs in the 0–10 cm and 10–20 cm soil depth layers of the 11 sampling locations (L1–L11) along the 20-km downwind transect away from the Almalyk copper smelter

The concentrations of Σ29 PAHs in the topsoils (0–10 cm) of the Almalyk area ranged from 55 to 2670 ng g^−1^. The highest concentration was found at location L1, which is a grassland next to the Cu processing factory (Figs. 1, 2; Table 1), while the lowest concentration was found at L3.

Locations (other than L1) with generally elevated concentrations of Σ29 PAHs (i.e. > 100 ng g^−1^) were L2 (223 ng g^−1^), L5 (182 ng g^−1^), L10 (113 ng g^−1^), and L4 (104 ng g^−1^). These data suggest that besides locations L1 and L2, at which the concentrations of PAHs in the soils were probably controlled by emissions of the Cu smelter and chemical factory, the concentrations of PAHs at other locations might be related to other anthropogenic activities (e.g., traffic or urban activities). The locations with elevated concentrations of Σ29 PAHs (i.e., L4, L5, and L10) were all grasslands and agricultural soils along the Almalyk-Buka highway (Table 1; Fig. 1), suggesting that traffic might also be an important source of PAH contamination at these sampling locations. The concentrations of several individual PAHs and ΣHMW-PAHs showed significant negative correlations with distance from the Almalyk Cu processing factory indicating that the factory is a source of the PAHs (Fig. S1).

**Fig. 3:**
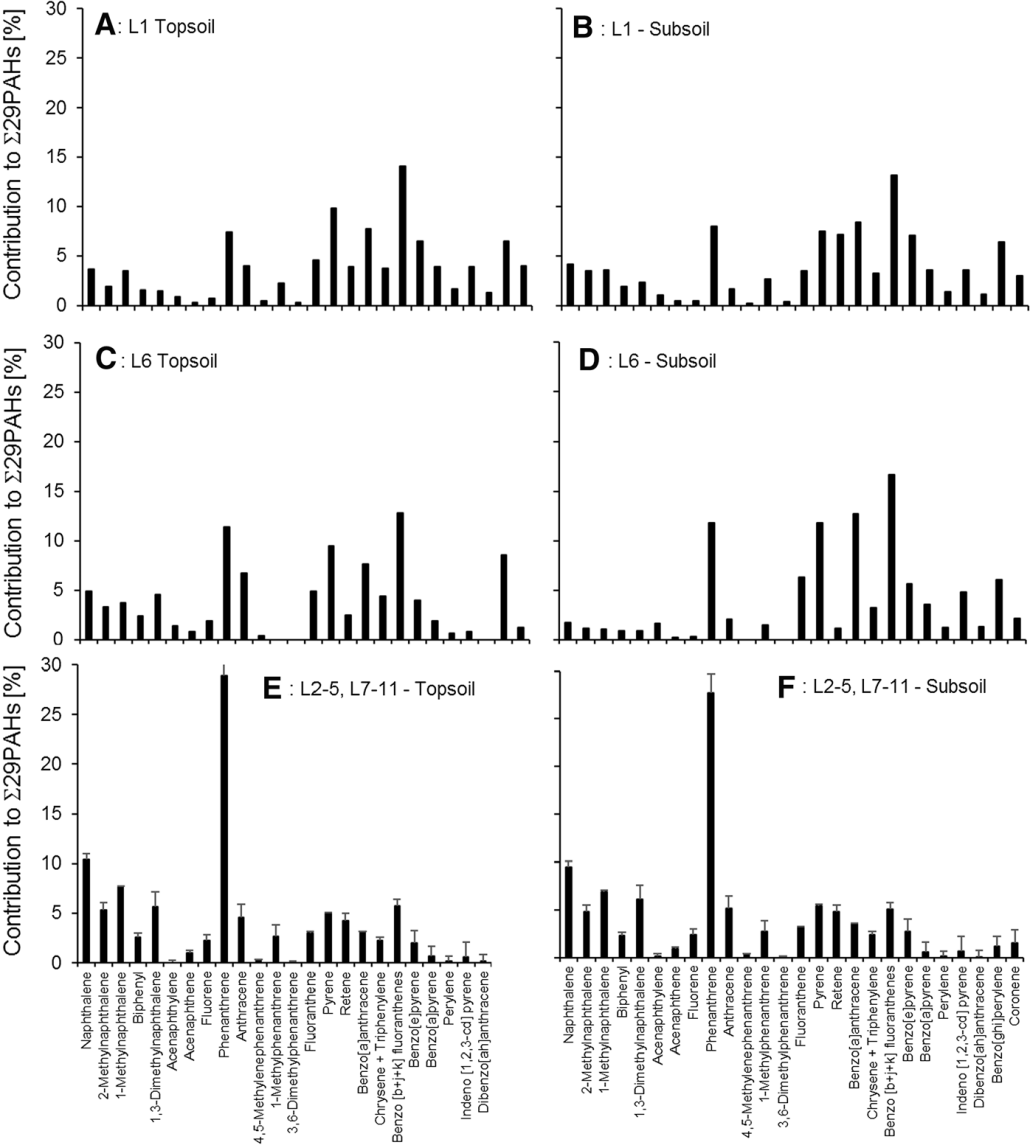
Contribution of individual PAHs to the Σ29 PAHs in **A** the topsoil of location L1, **B** the subsoil of L1, **C** the topsoil of L6, **D** the subsoil of L6, **E** the topsoils of L2-5 and L7-11 (mean of these sites), and **F** the subsoils of L2–5 and L7–11 (mean of these sites). Error bars show standard errors

**Fig. 4:**
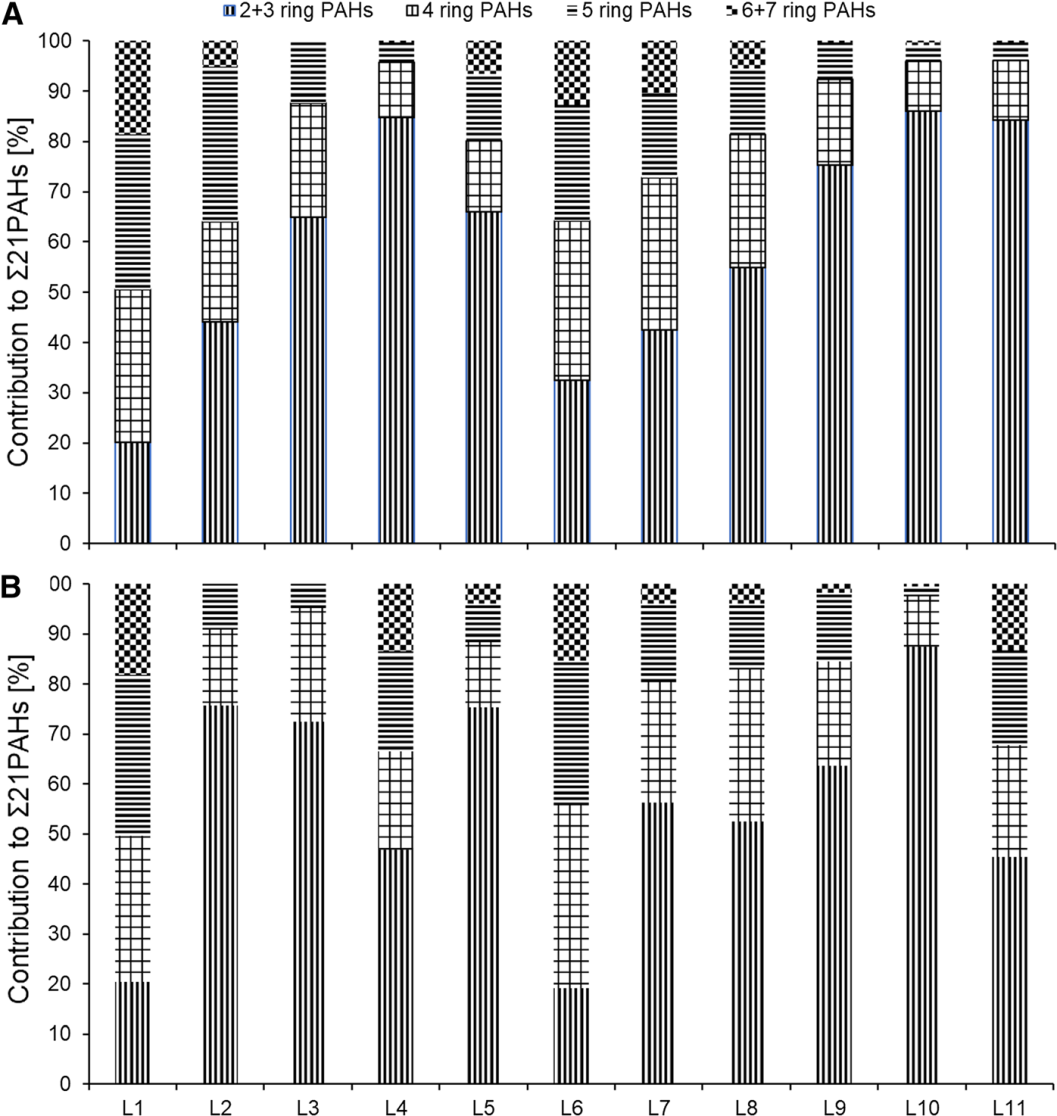
Contribution of the concentration of PAHs with different ring sizes to the concentration of Σ21 PAHs in **A** topsoils and **B** subsoils of Almalyk

The concentrations of the Σ29 PAHs in the deeper 10–20 cm layer ranged from 41 (L10) to 2180 ng g^−1^ (L1, Fig. 2). At most of the locations (L1, L2, L4, L7, L8 and L10), the concentrations of the Σ29 PAHs in the topmost 0–10 cm layer were higher than in the 10–20 cm depth layer. This indicates that atmospheric deposition was the main source of PAHs to the soils as already found elsewhere (Bandowe et al., 2010; Cousins, Beck, et al., 1999; Cousins, Gevao, et al., 1999). However, there were also several locations (L3, L5, L6, L9, and L11, Fig. 2) where the opposite was the case (i.e., the concentrations of PAHs in the 10–20 cm layer were higher than in the 0–10 cm layer). However, except at L6, the differences between the two layers were small (Fig. 2). We attribute this finding to soil mixing by plowing in arable fields, or biological activity in grasslands. Only at L6, the 10–20 cm layer showed a roughly 10 times higher Σ29 PAH concentration than the 0–10 cm layer (Fig. 2; Table S3), which might be a result of the burying of a contaminated previous surface soil by less contaminated soil material. This assumption is supported by the particularly low contribution of the 2–3 ring PAHs (19.1%) to the total sum of PAH concentrations, which was even smaller than in the 0–10 cm layer (32.5%, Fig. 4). Atmospheric deposition of PAHs on topsoil followed by leaching should result in higher contribution of the low-molecular weight PAHs to the sum of PAH concentrations in the subsoil than in the topsoil. We interpret the differences in the PAH mixtures in the 0–10 and 10–20 cm layers of L6 as the consequence of a contamination with different PAH mixtures (Figs. 3, 4). Location L6 is close to sedimentation tanks of a waste water treatment plant as well as a roadside cafeteria. We therefore speculate that the 0–10 cm layer at L6 might be a result of the deposition of little PAH-contaminated material from activities related with the sedimentation tanks.

Because most regulatory requirements refer to the concentration of the 16 US-EPA PAHs, most studies in the literature report only those values (Andersson and Achten, 2015). Hence, for comparison with other studies, we also show the concentration of ΣUS-EPA PAHs (Fig. 2). The spatial distribution of the concentration of ΣUS-EPA PAHs is similar to that of the Σ29 PAHs with the highest concentration (1940 ng g^−1^) in the 0–10 cm layer at L1, and the lowest at L3 (40 ng g^−1^, Fig. 2). In a previous study of a 20-km transect in and around the Angren industrial region of Uzbekistan (coal mine, coal power plant, rubber factory, gold refinery, including some background sites used for gardening, farming, grazing and as recreation area), we found higher concentrations of Σ31 PAHs (118–5190 ng g^−1^) and ΣUS-EPA PAHs (70–4230 ng g^−1^; Bandowe et al., 2010). The highest concentrations in the Angren soils occurred near a coal mine and associated power plant (Bandowe et al., 2010).

To the best of our knowledge, only two other studies have been published on PAH concentrations in central Asian soils. A study of mountain soils (0–5 cm) in Tajikistan reported concentrations of the ΣUS-EPA PAHs of 17–258 ng g^−1^ (Zhao et al., 2017), which is similar to what is found at most locations in Almalyk, except the highest concentrations next to the copper smelter (Table 2).

**Table 2:** Comparison of PAH concentrations in soils of Almalyk (this study) with PAH concentrations in soils reported in other studies

Location	Land use	Number of PAHs	Concentrations (ng g^−1^)	References
Almalyk, Uzbekistan	Industrial site (L1, 0–10 cm)	29	2670	This study
Almalyk, Uzbekistan	Industrial site (L1, 10–20 cm)	29	2180	This study
Almalyk, Uzbekistan	Other sites (L2–L11, 0–10 cm)	29	55–222	This study
Angren, Uzbekistan	Industrial to background transect (0–10 cm)	31	118–5910	Bandowe et al. (2010)
Angren, Uzbekistan	Industrial to background transect (10–20 cm)	31	104–3850	Bandowe et al. (2010)
Countrywide, Tajikistan	Diverse land use (0–5 cm)	16	17–258	Zhao et al. (2017)
Tian Shan Mountains, Kyrgyzstan	Diverse land use (0–5 cm)	16	52–9440	Li et al. (2020)
Countrywide, Poland	Arable soils (0–20 cm)	16	80–7264	Maliszewska-Kordybach et al. (2008)
NABO sites, Switzerland	Arable soils (0–20 cm)	16	219	Desaules et al. (2008)
NABO sites, Switzerland	Grasslands	16	106	Desaules et al. (2008)
Bayreuth, Germany	Highway traffic	20	814–4613	Glaser et al. (2005)
Jena, Germany	Grassland urban, city traffic	29	260–2400	Bandowe et al. (2019)
Northeast France	Highway traffic	16	1293	Crépineau-Ducoulombier and Rychen (2003)
Countrywide, South Korea	Agricultural soils	16	23–2830	Nam et al. (2003)
East China	Agricultural soils	20	8.8–3380	Sun et al. (2017)
Huanghui plain, China	Agricultural soils	16	16–1248	Yang et al. (2012)
Delhi, India	Agricultural soils	16	830–3880	Agarwal et al. (2009)
Transect native Prairie, North America	Grassland soils	20	63–321	Wilcke and Amelung (2000)
North–South transect, Argentina	Grassland background soils	29	2.4–38	Wilcke et al. (2014)
Mayabeque, Cuba	Urban, industrial, agricultural soils	16	20–106	Sosa et al. (2017)

Another recent study on PAH concentrations in soils (0–5 cm) of the Tian Shan Mountains of Kyrgyzstan reported ΣUS-EPA PAH concentrations of 52–9440 ng g^−1^ (Li et al., 2020). The soil samples with ΣUS-EPA PAH concentrations > 1000 ng g^−1^ were collected from industrial and urban sites (Li et al., 2020; Table 2). A second group of the soils of Kyrgyzstan had a ΣUS-EPA PAH concentrations range of 300–1000 ng g^−1^, and these soils were mainly from agricultural and rural regions (Li et al., 2020). The lowest concentrations of the ΣUS-EPA PAHs (52–299 ng g^−1^) in the Kyrgyzstan soils were mainly from pristine mountain regions (deserts, grassland and alpine meadows, Li et al., 2020). The concentrations of ΣUS-EPA PAHs in a majority of the Almalyk soils were in the range of those reported for pristine environments in Kyrgyzstan and the mountain soils of Tajikistan (Table 2; Li et al., 2020; Zhao et al., 2017). We therefore conclude that the operation of the copper smelter and chemical factory at Almalyk has not resulted in substantial pollution with PAHs in Almalyk soils beyond a 2-km radius (Table 2). The concentrations of PAHs in soils of several other geographic locations with similar land use as around Almalyk (agricultural fields, grasslands, soils near traffic sites, soils at industrial sites) reported in other studies are shown in Table 2. The concentrations of PAHs in the Almalyk soils fall in the range of the values reported in the soils with similar land-use (Table 2). The highest concentration of 2670 ng g^−1^ near Almalyk MMC was, however, lower than the highest concentration of PAHs in soils at industrial and traffic-influenced sites reported by other studies (Table 2).

In a classification system suggested for Agricultural soils of Poland, soils with ΣUS-EPA concentrations < 200 ng g^−1^, 200–600 ng g^−1^, 600–1000 ng g^−1^, and > 1000 ng g^−1^ were classified as “non-contaminated,” “weakly contaminated,” “contaminated” and “heavily “contaminated,” respectively (Maliszewska-Kordybach, 1996). Consequently, only location L1 and the 10–20 cm layer of location L6 can be classified as contaminated or weakly contaminated sites. Thus, PAH concentrations in Almalyk soils were mostly surprisingly low for a several decades-old heavy industrial area. They were for instance almost by a factor of 10 lower than in the A horizons of soils sampled in a forest near an aluminum smelter in Slovakia with Σ29 PAH concentrations of 2400–17,000 ng g^−1^ (Bandowe et al., 2018a) and lower than usually found in urban areas of the temperate zone (Wilcke, 2000). Forest soils generally receive and retain higher concentrations of semi-volatile organic compounds like PAHs than grassland soils because of the forest filter effect and high organic matter concentrations (Bandowe et al., 2019b; Wilcke, 2000) which might explain the higher concentrations in the Slovak than the Almalyk soils. Although it is possible that PAH emissions from the Almalyk MMC were lower than from the Slovak aluminum smelter, we speculate that there was a more pronounced (photo)chemical degradation of PAHs during transport in the atmosphere or on the surface of plants and soils after deposition as also suggested for tropical regions (Wilcke et al., 1999; Bandowe et al., 2014). Alternatively, the usually intense biological activity in the humid spring (Driessen and Deckers, 2001) could have resulted in a particularly pronounced PAH decomposition.

The composition of the PAH mixtures in environmental samples can be used for source reconnaissance, if potential transformations of the PAH mixtures during atmospheric transport and after deposition to soil are properly considered. The ΣHMW-/ΣLMW-PAH concentration ratios in Almalyk soil samples were markedly above unity at locations L1 (3.9 in the 0–10 cm, 3.8 in the 10–20 cm layers) and L6 (2.1 in the 0–10 cm, 4.5 in the 10–20 cm layers), but were < 1.0–1.5 at all other locations. This suggests that the PAH patterns at sites L1 and L6 were dominated by emissions of high-temperature combustion processes, which usually produce PAH mixtures with higher proportion of HMW-PAHs (i.e., PAHs with > 4 rings, Bandowe et al., 2010). This source attribution is supported by the fact that at locations L1 and L6, the PAH mixtures were dominated by the benzo[*b*+*j*+*k*]fluoranthenes (5-ring PAHs, Fig. 3).

At the other locations, the PAH patterns were dominated by phenanthrene, naphthalene and their alkylated derivatives, typical of background conditions (Fig. 3; Wilcke, 2007). Figure 4 further shows that the most volatile 2–3-ring PAHs (six compounds) contributed 52–87% to the concentrations of Σ21 parent-PAHs in 14 out of 22 soil samples.

This indicated that the sources of PAHs at these locations were atmospheric transport, biomass/vegetation burning, biogenic or petrogenic (Bandowe et al., 2018a, b; Wilcke and Amelung, 2000).

### Drivers of the vertical distribution of PAHs in Almalyk soils

The ratios of the PAH concentrations in topsoil (0–10 cm) to subsoil (10–20 cm) showed an inconsistent pattern (Table S3). The topsoil/subsoil concentration ratios of PAHs (CR) is indicative of the extent of contamination because of atmospheric input from anthropogenic sources and vertical translocation mechanisms in soil including leaching in dissolved form or associated with dissolved organic matter, bioturbation, plowing, or burying (Bandowe et al., 2010; Bandowe et al., 2011; Bandowe et al., 2019b; Krauss et al., 2000; Krauss and Wilcke, 2003). Our results suggest that several or all of the drivers of the vertical PAH distribution were at play in the studied soils. To further elucidate the role of leaching in explaining the vertical distribution of PAHs at our study sites, we correlated the log-transformed CR with the K_OW_ values of the PAHs (Table S4). There was no relationship (*p* > 0.05) between the logCR and logK_OW_ values at locations L1, L3, L8, L9 and L10. At locations L4 (*r* = − 0.69, *p* = 0.001), L6 (*r* = − 0.69, *p* < 0.001) and L11 (*r* = − 0.71, *p* = 0.001), the logCR were negatively correlated with logK_OW,_ values which might indicate that the main translocation mechanism was biological mixing or mechanical mixing by plowing, while the more soluble PAHs with low K_OW_ value were preferentially lost by biological degradation or volatilization. Significant positive correlations between the logCR and logK_OW_ values occurred at locations L2 (*r* = 0.52, *p* = 0.045), L5 (*r* = 0.72, *p* < 0.001) and L7 (*r* = 0.53, *p* = 0.027), indicating that leaching of dissolved PAHs played a role for the vertical distribution of PAHs at these three locations (Bandowe et al., 2011). We suggest that the comparatively high importance of mixing at the majority of the locations is related with low rainfall and seasonally strong bioturbation in the study region characteristic for semi-arid steppe regions.

### Relationship of PAH concentrations with soil properties including trace metal concentrations

The concentrations of phenanthrene (*r* = − 0.73, *p* = 0.011), retene (*r* = − 0.60, *p* = 0.05), and the ΣLMW-PAHs (*r* = − 0.61, *p* = 0.044) correlated significantly negative with the C_org_ concentrations (Fig. S2). This is in contrast to reported positive correlations of PAHs with C_org_ concentrations (Wilcke and Amelung, 2000). We can only speculate that this is related with enhanced volatilization of LMW-PAHs from soils with higher organic matter concentrations, because these soils retain soil moisture for a longer time than soils poor in organic matter. This in turn might favor volatilization at the high summer temperatures of our study area in spite of the fact that usually elevated *C*_org_ concentrations reduce volatilization. All other measured soil properties (pH, electrical conductivity and total nitrogen concentrations) did not correlate with the concentrations of any individual PAH or their sums.

Metal concentrations had earlier been measured in the same samples and reported by Shukurov et al. (2014). The previous study also identified emissions from the Almalyk metal mining and refining activities to have resulted in the enrichment of several trace metals in the soils with a negative impact on soil health (Shukurov et al., 2014). We found significant positive correlations between the total concentrations of Zn, Pb and Cu, and the concentrations of several individual PAHs (Figs. S3–S5a). If the highest Pb concentration was considered as an outlier and removed from our dataset, the correlations of Pb with PAH concentrations were no longer significant (Fig. S5b). The concentrations of Zn and Cu correlated with those of the ΣHMW-PAHs, while the concentration of Pb correlated with those of both ΣLMW-PAHs and ΣHMW-PAHs. In contrast, the concentrations of Cd, Cr and Ni were unrelated with any individual or sum of PAH concentration. The three metals that showed significant correlations with some PAHs are among those that are mined and refined at the Almalyk MMC (Shukurov et al., 2005, 2014). Our correlation analysis suggests that Zn, Cu, Pb and PAHs were emitted together from the stacks of the copper smelter during industrial operations. Metals and PAHs have (eco)toxicological properties and can therefore adversely impact the health of humans, livestock, wildlife and microrganisms. The co-occurrence of metals and PAHs can result in synergistic toxic effects (Gogolev and Wilke, 1997; Maliszewska-Kordybach and Smreczak, 2003; Muthusamy et al., 2016; Wang et al., 2015). Thus, the exposure of humans, livestock, wildlife, plants and microorganism to the Almalyk soils (which contain PAHs and toxic metals) can result in more severe adverse impacts because of possible synergistic effects. Furthermore, the presence of toxic metals can inhibit microbial diversity, enzyme activity and functions that might significantly reduce the dissipation of PAHs through microbial degradation (Sandrin and Maier, 2003; Thavamani et al., 2012).

### Risk and regulatory assessment of the PAH concentrations in soils

The concentrations of the Σ16 US-EPA PAHs in the soils at location L1 (next to the copper smelter) were the only ones that exceed the precautionary value of 1000 ng g^−1^ set in Switzerland (Desaules et al., 2008), while they were still far below the precautionary value of 10,000 ng g^−1^ set in Germany for soils with C concentrations < 8% (BBodSchV, 1999). The samples L1 (0–10 cm), L1 (10–20 cm) and L6 (10–20 cm) with the highest B[*a*]P TPE concentrations of 210, 161 and 74 ng g^−1^, respectively, were far below the Canadian threshold value of 600 ng g^−1^ for elevated risk in soils under agricultural, residential, parkland, commercial and industrial land use (CCME, 2010). All other sites had B[*a*]P TPE concentrations < 10 ng g^−1^. Thus, according to this assessment approach, none of the studied soils should pose an elevated cancer risk (CCME, 2010). However, the exposure of soil microorganisms, plants and humans to samples containing mixtures of PAHs and metals can result in synergistic toxic effects (Gogolev and Wilke, 1997; Maliszewska-Kordybach and Smreczak, 2003; Muthusamy et al., 2016; Thavamani et al., 2012; Wang et al., 2015). The occurrence of toxic metals in the PAH-contaminated soils can also result in diminished biodegradation of PAHs (Thavamani et al., 2012; Sandrin and Maier, 2003).

## Conclusions

Along a transect off Almalyk MMC, we found only one intermediately PAH-contaminated site next to the copper smelter, while PAH concentrations decreased to background values with increasing distance from the copper smelter. The contaminated soil was dominated by high-molecular weight PAHs likely originating from the combustion of fossil fuels, while the PAH mixtures in the low contaminated background soils were dominated by low molecular weight PAHs, particularly phenanthrene and retene.

The differences in PAH concentrations between the two soil depth layers at the 11 locations were generally small and inconsistent, except at one location where a former slightly contaminated surface appeared to be buried. The lack of a negative relationship of the topsoil/subsoil concentration ratios of PAHs with K_OW_ values supported our hypothesis that vertical PAH translocation occurred mainly by mixing, i.e., bioturbation or plowing, while leaching played a minor role in the semi-arid study area.

Surprisingly the concentrations of the LMW-PAHs correlated negatively with those of C_org_ concentrations. We suggest that this is related with increasing volatilization from soil with increasing organic matter concentrations, because organic matter-rich soil retains a sufficient soil moisture for volatilization for a longer time after the humid spring season. However, this speculation requires further experimental tests. Moreover, concentrations of Cu, Pb and Zn in soil correlated positively with many individual PAH concentrations indicating that the PAHs were emitted together with these trace metals by the copper smelter, resulting in a complex mixture of contaminants in the soils along the studied transect.

The comparison of the PAH concentrations in the Almalyk soils (this study) with the limit values set in the regulations of Switzerland and Germany (in the absence of specific Uzbek regulations) revealed that only one soil can be considered as contaminated with PAHs. The carcinogenic risk of the PAHs in this contaminated soil was below the limit value of Canada. Thus, the overall soil contamination with PAHs in the study area was surprisingly low, in spite of the several decades-long heavy industrial activities. This suggests a pronounced role of photochemical and microbial PAH degradation and volatilization preventing soil contamination in semi-arid regions similarly as previously reported from tropical sites.

## Supplementary Information

Below is the link to the electronic supplementary material.

Supplementary file 1 (DOCX 164 KB)

## Data Availability

All data and material for this manuscript can be obtained from the corresponding author upon request.

## References

[CR1] Achten C, Hofmann T (2009). Native polycyclic aromatic hydrocarbons (PAHs) in coals—A hardly recognized source of environmental contamination. Science of the Total Environment.

[CR2] Agarwal T, Khillare PS, Shridhar V, Ray S (2009). Pattern, sources, and toxic potential of PAHs in agricultural soils of Delhi, India. Journal of Hazardous Materials.

[CR3] Andersson JT, Achten C (2015). Time to say goodbye to the 16 EPA-PAHs? Toward an up-to-date use of PACs for environmental purposes. Polycyclic Aromatic Compounds.

[CR4] Arp HPH, Lundstedt S, Josefsson S, Cornelissen G, Enell A, Allard A-S, Kleja DB (2014). Native oxy-PAHs, N-PACs, and PAHs in historically contaminated soils from Sweden, Belgium, and France: Their soil-porewater partitioning behavior, bioaccumulation in *Enchytraeus crypticus*, and bioavailability. Environmental Science and Technology.

[CR5] Ashayeri NY, Keshavarzi B, Moore F, Kersten M, Yazdi M, Lahijanzadeh AR (2018). Presence of polycyclic aromatic hydrocarbons in sediments and surface water from Shadegan wetland—Iran: A focus on source apportionment, human and ecological risk assessment and sediment-water exchange. Ecotoxicology and Environmental Safety.

[CR6] Bandowe BAM, Bigalke M, Kobza J, Wilcke W (2018). Sources and fate of polycyclic aromatic compounds (PAHs, oxygenated PAHs and azaarenes) in forest soil profiles opposite of an aluminium plant. Science of the Total Environment.

[CR7] Bandowe BAM, Fränkl L, Grosjean M, Tylmann W, Mosquera PV, Hampel H, Schneider T (2018). A 150-year record of polycyclic aromatic compound (PAH) deposition from high Andean Cajas National Park, Ecuador. Science of the Total Environment.

[CR8] Bandowe BAM, Leimer S, Meusel H, Velescu A, Dassen S, Eisenhauer N, Hoffmann T, Oelmann Y, Wilcke W (2019). Plant diversity enhances the natural attenuation of polycyclic aromatic compounds (PAHs and oxygenated PAHs) in grassland soils. Soil Biology and Biochemistry.

[CR9] Bandowe BAM, Shukurov N, Kersten M, Wilcke W (2010). Polycyclic aromatic hydrocarbons (PAHs) and their oxygen-containing derivatives (OPAHs) in soils from the Angren industrial area, Uzbekistan. Environmental Pollution.

[CR10] Bandowe BAM, Sobocka J, Wilcke W (2011). Oxygen-containing polycyclic aromatic hydrocarbons (OPAHs) in urban soils of Bratislava, Slovakia: Patterns, relation to PAHs and vertical distribution. Environmental Pollution.

[CR11] Bandowe BAM, Wei C, Han YM, Cao JJ, Zhan C, Wilcke W (2019). Polycyclic aromatic compounds (PAHs, oxygenated PAHs, nitrated PAHs and azaarenes) in soils from China and their relationship with geographic location, land use and soil carbon fractions. Science of the Total Environment.

[CR12] Bandowe BAM, Wilcke W (2010). Analysis of polycyclic aromatic hydrocarbons and their oxygen-containing derivatives and metabolites in soils. Journal of Environmental Quality.

[CR13] BbodSchV. (1999). Bundes-Bodenschutz- und Altlastenverordnung vom 12. Juli 1999. BGBl. I, S., Berlin (in German).

[CR14] Cai QY, Mo CH, Wu QT, Katsoyiannis A, Zeng QY (2008). The status of soil contamination by semi-volatile organic compounds (SVOCs) in China: A review. Science of the Total Environment.

[CR15] CCME [Canadian Council of Ministers of the Environment]. (2010). Canadian soil quality guidelines for carcinogenic and other polycyclic aromatic hydrocarbons (Environmental and Human Health Effects). Scientific Criteria Document (revised) PN 1445, Quebec, Canada. https://www.ccme.ca/files/Resources/supporting_scientific_documents/pah_soqg_scd_1445.pdf. Accessed January 2020.

[CR16] Cousins IT, Beck AJ, Jones KC (1999). A review of the processes involved in the exchange of semi-volatile organic compounds (SVOC) across the air–soil interface. Science of the Total Environment.

[CR17] Cousins IT, Gevao B, Jones KC (1999). Measuring and modelling the vertical distribution of semi-volatile organic compounds in soils. I. PCB and PAH soil core data. Chemosphere.

[CR18] Crépineau-Ducoulombier C, Rychen G (2003). Assessment of soil and grass polycyclic aromatic hydrocarbon (PAH) contamination in agricultural fields near a motorway and an airport. Agronomie.

[CR19] Davie-Martin CL, Stratton KG, Teeguarden JG, Waters KM, Simonich SLM (2017). Implications of bioremediation for polycyclic aromatic hydrocarbon contaminated soils for human health and cancer risk. Environmental Science and Technology.

[CR20] De Laender F, Hammer J, Hendriks AJ, Soetaert K, Janssen CR (2011). Combining monitoring data and modeling identifies PAHs as emerging contaminants in the Arctic. Environmental Science and Technology.

[CR21] Desaules A, Ammann S, Blum F, Brändli RC, Bucheli TD, Keller A (2008). PAHs and PCBs in soils of Switzerland—Status and critical review. Journal of Environmental Monitoring.

[CR22] Douben PET (2003). PAHs: an ecotoxicological perspective. Wiley, New York.

[CR23] Driessen P, Deckers J (2001). Lecture notes on the major soils of the world.

[CR59] Encyclopaedia Britannica. (2018). Olmaliq. https://www.britannica.com/place/Olmaliq. Accessed 16 November 2020.

[CR24] Gogolev A, Wilke B-M (1997). Combination effects of heavy metals and fluoranthene on soil bacteria. Biology and Fertility of Soils.

[CR25] Gross, J., & Ligges, U. (2015). Nortest: Tests for normality. R package version 1.0-4. https://CRAN.R-project.org/package=nortest. Accessed January 2020.

[CR26] IARC (2010). Some non-heterocyclic polycyclic aromatic hydrocarbons and some related exposures. IARC Monographs on the Evaluation of Carcinogenic Risks to Humans.

[CR27] IUSS Working Group WRB. (2014). World reference base for soil resources 2014. In *International soil classification system for naming soils and creating legends for soil maps*. World Soil Resource Reports No. 106. FAO, Rome. http://www.fao.org.

[CR28] Katsoyiannis A, Sweetman AJ, Jones KC (2011). PAH molecular diagnostic ratios applied to atmospheric sources: a critical evaluation using two decades of source inventory and air concentration data from the UK. Environmental Science and Technology.

[CR29] Keyte IJ, Harrison RM, Lammel G (2013). Chemical reactivity and long-range transport potential of polycyclic aromatic hydrocarbons—A review. Chemical Society Reviews.

[CR30] Kjeller L-O (1998). Addition of internal standards to particulate sample matrices for routine trace analysis of semi volatile organic compounds: A source of systematical and random errors. Fresenius Zeitschrift Für Analytische Chemie.

[CR31] Kodirov O, Shukurov N (2009). Heavy metal distribution in soils near the Almalyk mining and smelting industrial area, Uzbekistan. Acta Geologica Sinica.

[CR32] Krauss M, Wilcke W (2003). Polychlorinated naphthalenes in urban soils: analysis, concentrations, and relation to other persistent organic pollutants. Environmental Pollution.

[CR33] Krauss M, Wilcke W, Zech W (2000). Polycyclic aromatic hydrocarbons and polychlorinated biphenyls in forest soils: Depth distribution as indicator of different fate. Environmental Pollution.

[CR34] Li Q, Wu J, Zhou J, Sakiev K, Hofmann D (2020). Occurrence of polycyclic aromatic hydrocarbon (PAH) in soils around two typical lakes in the western Tian Shan Mountains (Kyrgyzstan, Central Asia): Local burden or global distillation?. Ecological Indicators.

[CR35] Lima ALC, Farrington JW, Reddy CM (2005). Combustion-derived polycyclic aromatic hydrocarbons in the environment. Environmental Forensics.

[CR36] Maliszewska-Kordybach B (1996). Polycyclic aromatic hydrocarbons in agricultural soils in Poland: Preliminary proposals for criteria to evaluate the level of soil contamination. Applied Geochemistry.

[CR37] Maliszewska-Kordybach B, Smreczak B (2003). Habitat function of agricultural soils as affected by heavy metals and polycyclic aromatic hydrocarbons contamination. Environment International.

[CR38] Maliszewska-Kordybach B, Smreczak B, Klimkowicz-Pawlas A, Terelak H (2008). Monitoring of the total content of polycyclic aromatic hydrocarbons (PAHs) in arable soils in Poland. Chemosphere.

[CR40] Muntean N, Jermini M, Small I, Falzon D, Fürst P, Migliorati G, Scortichini G, Forti AF, Anklam E, von Holst C, Niyazmatov B, Bahkridinov S, Aertgeerts R, Bertollini R, Tirado C, Kolb A (2003). Assessment of dietary exposure to some persistent organic pollutants in the Republic of Karakalpakstan of Uzbekistan. Environmental Health Perspectives.

[CR41] Muthusamy S, Peng S, Ng JC (2016). The binary, ternary and quaternary mixture toxicity of benzo[*a*]pyrene, arsenic, cadmium and lead in HepG2 cells. Toxicology Research.

[CR42] Nam JJ, Song BH, Eom KC, Lee SH, Smith A (2003). Distribution of polycyclic aromatic hydrocarbons in agricultural soils in South Korea. Chemosphere.

[CR44] Neff JM, Stout SA, Gunstert DG (2005). Ecological risk assessment of polycyclic aromatic hydrocarbons in sediments: Identifying sources and ecological hazard. Integrated Environmental Assessment and Management.

[CR45] Pen-Mouratov S, Shukurov N, Steinberger Y (2008). Influence of industrial heavy metal pollution on soil free-living nematode population. Environmental Pollution.

[CR47] R Core Team. (2019). *R: A language and environment for statistical computing*. R Foundation for Statistical Computing.

[CR49] Rowell, D. L. (1994). *Soil science: Methods and applications*. Longmans Group UK Ltd.

[CR50] Safirova, E. (2019). The mineral industry of Uzbekistan. U.S. Geological Survey, 2015 Minerals Yearbook. https://prd-wret.s3-us-west-2.amazonaws.com/assets/palladium/production/atoms/files/myb3-2015-uz.pdf. Date accessed November 16, 2020.

[CR51] Sandrin TR, Maier RM (2003). Impact of metals on the biodegradation of organic pollutants. Environmental Health Perspectives.

[CR52] Sharov P, Dowling R, Gogishvili M, Jones B, Caravonas J, McCartor A, Kashdan Z, Fuller R (2016). The prevalence of toxic hotspots in former Soviet countries. Environmental Pollution.

[CR53] Shukurov N, Kodirov O, Peitzch M, Kersten M, Pen-Mouratov S, Steinberger Y (2014). Coupling geochemical, mineralogical and microbial approaches to assess the health of contaminated soil around the Almalyk mining and smelter complex, Uzbekistan. Science of the Total Environment.

[CR54] Shukurov N, Pen-Mouratov S, Steinberger Y (2005). The impact of Almalyk industrial complex on soil chemical and biological properties. Environmental Pollution.

[CR55] Sosa D, Hilber I, Faure R, Bartolome N, Fonseca O, Keller A, Schwab P, Escobar A, Bucheli TD (2017). Polycyclic aromatic hydrocarbons and polychlorinated biphenyls in soils of Mayabeque, Cuba. Environmental Science and Pollution Research.

[CR56] Sun J, Pan L, Tsang DCW, Zhan Y, Zhu L, Li X (2018). Organic contamination and remediation in the agricultural soils of China: A critical review. Science of the Total Environment.

[CR57] Sun Z, Liu J, Zhuo S, Chen Y, Zhang Y, Shen H, Zeng EY (2017). Occurrence and geographic distribution of polycyclic aromatic hydrocarbons in agricultural soils in Eastern China. Environmental Science and Pollution Research.

[CR58] Thavamani P, Malik S, Beer M, Megharaj M, Naidu R (2012). Microbial activity and diversity in long-term mixed contaminated soils with respect to polyaromatic hydrocarbons and heavy metals. Journal of Environmental Management.

[CR60] Tobiszewski M, Namiesnik J (2012). PAH diagnostic ratios for the identification of pollution emission sources. Environmental Pollution.

[CR61] UNECE [United Nations Economic Commission for Europe]. (2001a). Environmental Performance Reviews. Uzbekistan. 1st review, UN, New York, Geneva, 2001. http://www.unece.org/fileadmin/DAM/env/epr/epr_studies/uzbekistan%20e.pdf. Accessed January 2020.

[CR62] UNECE [United Nations Economic Commission for Europe]. (2001b). 1st Environmental performance review Uzbekistan. Environmental performance reviews series no. 14. New York and Geneva: United Nations; 2001. http://www.unece.org/fileadmin/DAM/env/epr/epr_studies/uzbekistan%20e.pdf. Date accessed November 16, 2020.

[CR63] UNECE [United Nations Economic Commission for Europe]. (2010). Environmental Performance Reviews. Uzbekistan. Second review, UN, New York, Geneva, 2010. http://www.unece.org/fileadmin/DAM/env/epr/epr_studies/uzbekistan%20II%20e.pdf. Accessed January 2020.

[CR64] Wang T, Feng W, Kuang D, Deng Q, Zhang W, Wang S, He M, Zhang X, Wu T, Guo H (2015). The effects of heavy metals and their interactions with polycyclic aromatic hydrocarbons on the oxidative stress among coke-oven workers. Environmental Research.

[CR65] Wilcke W (2000). Polycyclic aromatic hydrocarbons in soil—a review. Journal of Plant Nutrition and Soil Science.

[CR66] Wilcke W (2007). Global patterns of polycyclic aromatic hydrocarbons (PAHs) in soil. Geoderma.

[CR67] Wilcke W, Amelung W (2000). Persistent organic pollutants in native grassland soils along a climosequence in North America. Soil Science Society Journal of America.

[CR68] Wilcke W, Bandowe BAM, Lueso MG, Ruppenthal M, del Valle H, Oelmann Y (2014). Polycyclic aromatic hydrocarbons (PAHs) and their polar derivatives (oxygenated PAHs, azaarenes) in soils along a climosequence in Argentina. Science of the Total Environment.

[CR69] Wilcke W, Kiesewetter M, Bandowe BAM (2014). Microbial formation and degradation of oxygen-containing polycyclic aromatic hydrocarbons (OPAHs) in soil during short-term incubation. Environmental Pollution.

[CR70] Wilcke W, Müller S, Kanchanakool N, Niamskul C, Zech W (1999). Polycyclic aromatic hydrocarbons (PAHs) in hydromorphic soils of the tropical metropolis Bangkok. Geoderma.

[CR71] Wild SR, Jones KC (1995). Polynuclear aromatic hydrocarbons in the United Kingdom environment. Environmental Pollution.

[CR72] Zhao Z, Zeng H, Wu J, Zhang L (2017). Concentrations, sources and potential ecological risks of polycyclic aromatic hydrocarbons in soils of Tajikistan. International Journal of Environment and Pollution.

