# Supplementary material for: Polycyclic aromatic hydrocarbons (PAHs) in soils of an industrial area in semi-arid Uzbekistan: spatial distribution, relationship with trace metals and risk assessment
Source: Environ Geochem Health. 2021 May 26;43(11):4847–61. doi: 10.1007/s10653-021-00974-3 (PMC8528758; doi:10.1007/s10653-021-00974-3)
Supplement: Supplementary file 1 — (DOCX 164 KB) [file 10653_2021_974_MOESM1_ESM.docx]

**Supplementary Information**

**Polycyclic aromatic hydrocarbons (PAHs) in soils of an industrial area in semi-arid Uzbekistan: spatial distribution, relationship with trace metals, and risk assessment**

Benjamin A. Musa Bandowe^a*^, Nosir Shukurov^b,c^, Sophia Leimer^d^, Michael Kersten^c^, Yosef Steinberger^e^, Wolfgang Wilcke^d^

^a^ Multiphase Chemistry Department, Max-Planck Institute for Chemistry, Hahn-Meitner-Weg 1, 55128 Mainz, Germany.

^b^ Institute of Geology and Geophysics, State committee of the Republic of Uzbekistan for Geology and Mineral Resources, Olimlar street 49, Tashkent 100041, Uzbekistan.

^c^ Geosciences Institute, Johannes Gutenberg-University, 55099 Mainz, Germany.

^d^ Institute of Geography and Geoecology, Karlsruhe Institute of Technology (KIT), Reinhard-Baumeister-Platz 1, 76131 Karlsruhe, Germany.

^e^ The Mina and Everard Goodman Faculty of Life Sciences, Bar-Ilan University, Ramat-Gan 52900, Israel.

*Corresponding author: Benjamin A. Musa Bandowe (E-mail: benjamin.bandowe@mpic.de; Tel.: + 49 6131 305 7061).

Supplementary Information contains Tables S1-S4 and Figures S1-S5.

| **Table S1** Mean recoveries of the target PAHs in the certified reference soil material (ERM-CC013a-Polycyclic aromatic hydrocarbons in soil) during the period in which the analyses of the Almalyk soil samples were analyzed.   \|  \| Certified value or indicative values \| Uncertainty \| Mean of recovery [%] \| \| --- \| --- \| --- \| --- \| \|  \|  \|  \|  \| \| PAHs \| mass fraction (ng/g) \| ± in ng/g \|  \| \| Naphthalene \| 2400 \| 500 \| 114 \| \| Acenaphthylene^a^ \| 770 \|  \| 82 \| \| Acenaphthene^a^ \| 750 \|  \| 94 \| \| Fluorene \| 1140 \| 110 \| 101 \| \| Phenanthrene \| 12000 \| 600 \| 106 \| \| Anthracene \| 1410 \| 220 \| 144 \| \| Fluoranthene \| 12900 \| 700 \| 46 \| \| Pyrene \| 9600 \| 300 \| 108 \| \| Benzo[a]anthracene \| 5600 \| 500 \| 119 \| \| Chrysene^b^ \| 5300 \| 800 \| 72 \| \| Benzo[b+k]fluoranthene^c^ \| 10400 \| 1400 \| 115 \| \| Benzo[a]pyrene \| 4900 \| 700 \| 85 \| \| Indeno[1,2,3-cd]pyrene \| 5200 \| 1000 \| 75 \| \| Dibenzo[a,h]anthracene^a^ \| 1100 \|  \| 106 \| \| Benzo[g,h,i]perylene \| 4600 \| 500 \| 94 \|   a: indicative values (not certified)  b: values from our laboratory measurement is the sum of Chrysene and Tripheylene  c: values from our laboratory measurement is the sum of Benzo[b+j+k]fluoranthene  **Table S2** Data transformations to approximate normal distribution. For variables marked with an asterisk, only an approximate normal distribution was reached by transformation. | | |
| --- | --- | --- | --- | --- | --- | --- | --- | --- | --- | --- | --- | --- | --- | --- | --- | --- | --- | --- | --- | --- | --- | --- | --- | --- | --- | --- | --- | --- | --- | --- | --- | --- | --- | --- | --- | --- | --- | --- | --- | --- | --- | --- | --- | --- | --- | --- | --- | --- | --- | --- | --- | --- | --- | --- | --- | --- | --- | --- | --- | --- | --- | --- | --- | --- | --- | --- | --- | --- | --- | --- | --- | --- | --- | --- |
|  | Transformation for 0-10 cm | Transformation for 10-20 cm |
| Total Zn (mg/kg) | -1/x | -1/x |
| Total Cu (mg/kg) | log(x) | log(x) |
| Total Pb (mg/kg) | log(x) | -1/x |
| Total Cd (mg/kg) | -1/x | -1/(x+1) |
| Total Cr (mg/kg) | log(x) | log(x) |
| Total Ni (mg/kg) | none | none |
| C_org_ (mg/g) | none | -1/x |
| Total soluble nitrogen (mg/l) | none | none |
| Soil moisture (%) | none | log(x) |
| pH | none* | none* |
| Electrical conductivity (mS/g) | log(x) | log(x)* |
| Naphthalene (ng/g) | log(x) | log(x) |
| 2-Methylnaphthalene (ng/g) | log(x) | log(x) |
| 1-Methylnaphthalene (ng/g) | log(x) | log(x) |
| Biphenyl (ng/g) | log(x) | log(x) |
| 1,3-Dimethylnaphthalene (ng/g) | log(x) | log(x) |
| Acenaphthene (ng/g) | log(x+1) | log(x+1) |
| Fluorene (ng/g) | log(x) | log(x) |
| Phenanthrene (ng/g) | log(x) | log(x) |
| Anthracene (ng/g) | log(x) | log(x) |
| 4,5-Methylenephenanthrene (ng/g) | -1/(x+1) | excluded |
| 1-Methylphenanthrene (ng/g) | excluded | log(x+1) |
| Fluoranthene (ng/g) | -1/x | -1/x |
| Pyrene (ng/g) | -1/x | log(x)* |
| Benzo[a]anthracene (ng/g) | log(x) | -1/(x+1) |
| Chrysene+Triphenylene (ng/g) | -1/x | -1/(x+1) |
| Benzo[b+j+k]fluoranthene (ng/g) | log(x) | log(x)* |
| Benzo[e]pyrene (ng/g) | -1/(x+1) | log(x) |
| Indeno[1,2,3-cd]pyrene (ng/g) | -1/(x+1) | -1/(x+1) |
| Coronene (ng/g) | excluded | log(x+1) |
| Σ 29PAHs (ng/g) | -1/x | log(x) |
| Σ21 Parent-PAHs (ng/g) | -1/x* | -1/x |
| ΣUS-EPA PAHs (ng/g) | -1/x | -1/x |
| ΣLMW-PAHs (ng/g) | log(x) | log(x) |
| ΣHMW-PAHs (ng/g) | log(x) | log(x)* |
| Σ2+3 ring PAHs (ng/g) | log(x) | log(x) |
| Σ4 ring PAHs (ng/g) | -1/x | log(x)* |
|  | | |
| **Table S2** Continued | | |
| Σ5 ring PAHs (ng/g) | log(x) | log(x)* |
| Σ6+7-ring PAHs (ng/g) | log(x+1) | log(x+1) |
| Σalkyl-Naphthalene (ng/g) | log(x) | log(x) |
| Retene (ng/g) | -1/x | -1/x |
| ΣAlkyl-Phenanthrene (ng/g) | log(x+1) | log(x+1) |

**Table S3** Topsoil/subsoil concentration ratios (CR) of individual and sums of PAHs.

|  | |  |  |  |  |  |  |  |  |  |  |  |  | |
| --- | --- | --- | --- | --- | --- | --- | --- | --- | --- | --- | --- | --- | --- | --- |
| CR (0-10 cm/10-20 cm) |  | |  |  |  |  |  |  |  |  |  |  |  |  |
| Study site |  | | L1 | L2 | L3 | L4 | L5 | L6 | L7 | L8 | L9 | L10 | L11 |  |
| Naphthalene |  | | 1.07 | 1.22 | 1.80 | 1.27 | 0.65 | 0.27 | 0.67 | 0.86 | 1.02 | 8.67 | 1.24 |  |
| 2-Methylnaphthalene |  | | 0.65 | 1.29 | 2.65 | 1.49 | 0.66 | 0.29 | 0.86 | 1.09 | 0.88 | 6.61 | 1.22 |  |
| 1-Methylnaphthalene |  | | 1.19 | 1.07 | 1.49 | 1.51 | 0.74 | 0.33 | 0.54 | 0.69 | 1.84 | 6.14 | 1.92 |  |
| Biphenyl |  | | 1.04 | 3.19 | 0.87 | 2.20 | 0.62 | 0.26 | 2.52 | 3.12 | 0.24 | 6.15 | 0.61 |  |
| 1,3-Dimethylnaphthalene |  | | 0.79 | 0.98 | 0.60 | 1.17 | 0.92 | 0.49 | 0.77 | 1.64 | 0.89 | 3.45 | 1.06 |  |
| Acenaphthylene |  | | 0.99 |  | 0.00 |  |  | 0.08 |  |  |  |  |  |  |
| Acenaphthene |  | | 0.91 |  |  | 1.62 | 0.99 | 0.33 |  | 1.69 | 0.00 | 2.31 | 0.81 |  |
| Fluorene |  | | 1.69 | 2.23 | 1.80 | 1.01 | 1.04 | 0.62 | 1.33 | 1.64 | 0.67 | 1.34 | 0.53 |  |
| Phenanthrene |  | | 1.13 | 0.80 | 0.84 | 1.60 | 1.06 | 0.09 | 0.93 | 1.99 | 1.35 | 1.80 | 1.35 |  |
| Anthracene |  | | 2.89 | 0.85 | 0.13 | 17.21 | 0.46 | 0.31 | 0.96 | 1.75 | 0.54 | 1.60 | 3.36 |  |
| 4H-Cyclopenta[d,e,f]phenanthrene |  | | 2.62 |  |  |  | 0.95 |  |  | 0.00 | 0.84 | 1.12 | 0.82 |  |
| 1-Methylphenanthrene |  | | 1.02 |  | 0.00 | 1.57 | 0.97 | 0.00 |  |  | 0.88 | 1.30 | 1.09 |  |
| 3,6-Dimethylphenanthrene |  | | 0.91 |  |  |  |  |  |  |  |  |  |  |  |
| Fluoranthene |  | | 1.61 | 1.52 | 0.58 | 1.36 | 1.18 | 0.08 | 1.42 | 1.46 | 0.91 | 1.63 | 0.99 |  |
| Pyrene |  | | 1.59 | 1.46 | 0.80 | 0.86 | 1.24 | 0.08 | 1.43 | 1.16 | 0.89 | 3.04 | 0.46 |  |
| Retene |  | | 0.66 | 1.21 | 0.57 | 1.44 | 0.86 | 0.20 | 1.49 | 2.24 | 0.84 | 0.99 | 0.99 |  |
| Benzo[a]anthracene |  | | 1.12 | 4.63 | 1.29 | 0.19 | 0.61 | 0.06 | 2.16 | 2.39 | 0.33 | 3.41 | 0.16 |  |
| Chrysene + Triphenylene |  | | 1.40 | 2.37 | 1.23 | 0.42 | 1.60 | 0.13 | 1.25 | 1.09 | 1.09 | 2.32 | 0.32 |  |
| Benzo[b+j+k]fluoranthenes |  | | 1.31 | 5.08 | 2.54 | 0.39 | 1.64 | 0.08 | 1.11 | 1.49 | 0.55 | 5.60 | 0.21 |  |
| Benzo[e]pyrene |  | | 1.12 | 5.45 | 1.40 | 0.09 | 1.65 | 0.07 | 1.55 | 1.41 | 0.55 | 0.00 | 0.11 |  |
| Benzo[a]pyrene |  | | 1.33 |  |  | 0.15 | 1.85 | 0.05 |  |  | 0.00 | 0.00 | 0.00 |  |
| Perylene |  | | 1.43 |  |  | 0.31 | 2.05 | 0.05 |  |  |  |  | 0.00 |  |
| Indeno [1,2,3-cd] pyrene |  | | 1.34 |  |  | 0.12 | 2.76 | 0.02 | 2.18 | 0.70 | 0.71 | 3.51 | 0.08 |  |
| Dibenzo[ah]anthracene |  | | 1.34 |  |  |  |  | 0.00 |  |  |  |  | 0.00 |  |
| Benzo[ghi]perylene |  | | 1.24 |  |  | 0.00 | 1.53 | 0.14 |  |  |  |  | 0.00 |  |
| Coronene |  | | 1.61 |  |  | 0.00 | 1.43 | 0.05 | 1.46 | 0.94 | 0.00 |  | 0.00 |  |
| ∑29 PAHs |  | | 1.22 | 1.56 | 0.82 | 1.13 | 0.90 | 0.10 | 1.17 | 1.65 | 0.93 | 2.74 | 0.85 |  |
| Σ21 Parent PAHs |  | | 1.33 | 1.73 | 0.83 | 1.02 | 0.99 | 0.09 | 1.23 | 1.64 | 0.92 | 2.52 | 0.73 |  |
| ΣUS EPA PAHs |  | | 1.33 | 1.62 | 0.82 | 1.20 | 0.96 | 0.09 | 1.22 | 1.67 | 0.95 | 2.53 | 0.82 |  |
| ΣLMW-PAHs |  | | 1.30 | 1.01 | 0.75 | 1.84 | 0.87 | 0.15 | 0.93 | 1.72 | 1.09 | 2.48 | 1.36 |  |
| ΣHMW-PAHs |  | | 1.33 | 3.89 | 1.06 | 0.29 | 1.36 | 0.07 | 1.62 | 1.55 | 0.63 | 2.77 | 0.21 |  |
|  | |  |  |  |  |  |  |  |  |  |  |  |  |  |

**Table S4** Results of Pearson correlations between log K_OW_ values and the respective log concentration ratios (CR).

| Concentration ratio (CR) | p | r |
| --- | --- | --- |
| Log L1 (10-20 cm/0-10 cm) | 0.568 | -0.12 |
| Log L1 (0-10 cm/10-20 cm) | 0.568 | 0.12 |
| Log L2 (10-20 cm/0-10 cm) | 0.045 | -0.52 |
| Log L2 (0-10 cm/10-20 cm) | 0.045 | 0.52 |
| Log L3 (10-20 cm/0-10 cm) | 0.882 | 0.04 |
| Log L3 (0-10 cm/10-20 cm) | 0.882 | -0.04 |
| Log L4 (10-20 cm/0-10 cm) | 0.001 | 0.69 |
| Log L4 (0-10 cm/10-20 cm) | 0.001 | -0.69 |
| Log L5 (10-20 cm/0-10 cm) | <0.001 | -0.72 |
| Log L5 (0-10 cm/10-20 cm) | <0.001 | 0.72 |
| Log L6 (10-20 cm/0-10 cm) | <0.001 | 0.69 |
| Log L6 (0-10 cm/10-20 cm) | <0.001 | -0.69 |
| Log L7 (10-20 cm/0-10 cm) | 0.027 | -0.53 |
| Log L7 (0-10 cm/10-20 cm) | 0.027 | 0.53 |
| Log L8 (10-20 cm/0-10 cm) | 0.671 | 0.11 |
| Log L8 (0-10 cm/10-20 cm) | 0.671 | -0.11 |
| Log L9 (10-20 cm/0-10 cm) | 0.431 | 0.20 |
| Log L9 (0-10 cm/10-20 cm) | 0.431 | -0.20 |
| Log L10 (10-20 cm/0-10 cm) | 0.194 | 0.32 |
| Log L10 (0-10 cm/10-20 cm) | 0.194 | -0.32 |
| Log L11 (10-20 cm/0-10 cm) | 0.001 | 0.71 |
| Log L11 (0-10 cm/10-20 cm) | 0.001 | -0.71 |

| 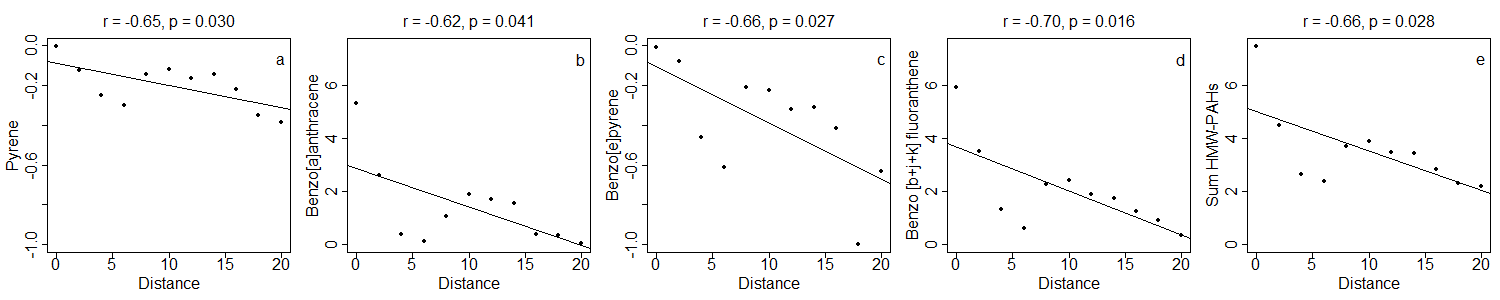 |
| --- |

**Fig. S1** Relationships between distance from the Almalyk Cu smelter [km] and concentrations of PAHs [ng g^-1^].


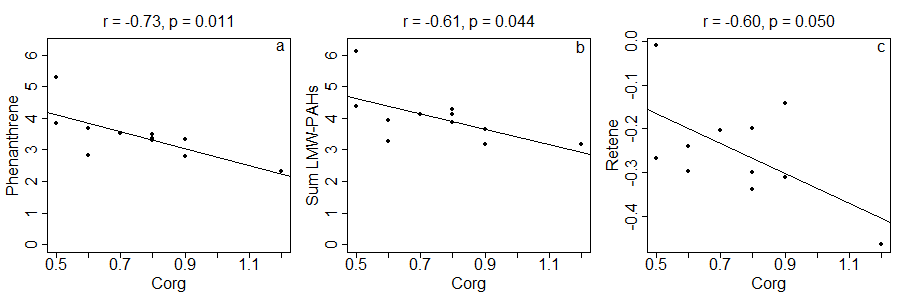


**Fig. S2** Relationships between concentration of soil organic C [Corg, mg g^-1^] and PAHs [ng g^-1^].


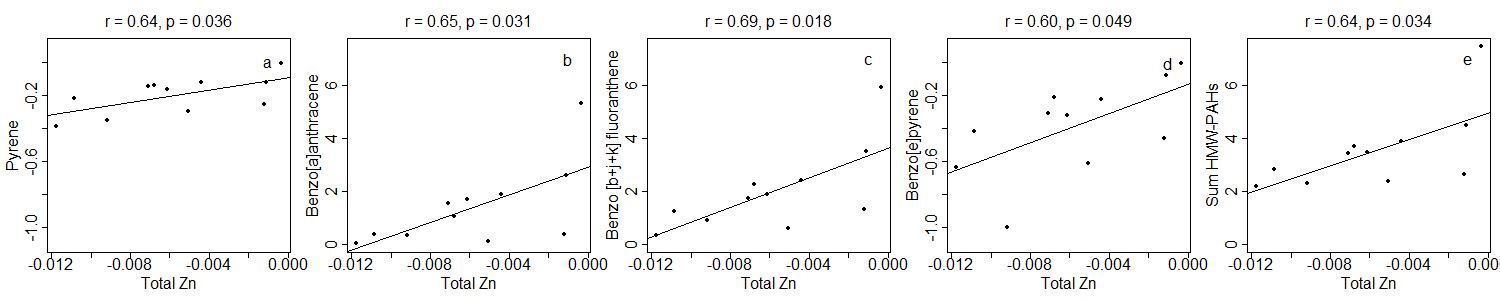


**Fig. S3** Relationships between concentration of Zn [µg g^-1^] and PAHs [ng g^-1^].


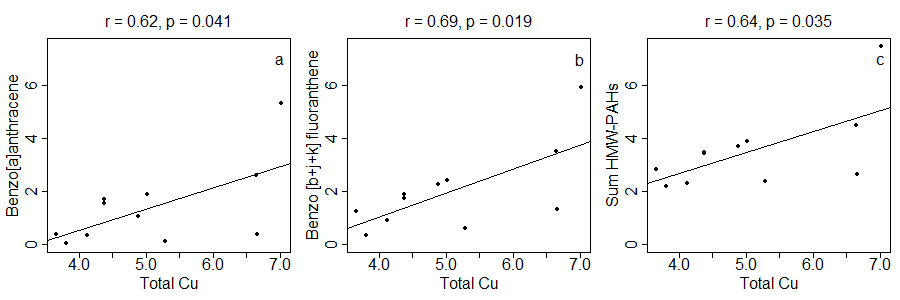


**Fig. S4** Relationships between concentration of Cu [µg g^-1^] and PAHs [ng g^-1^].


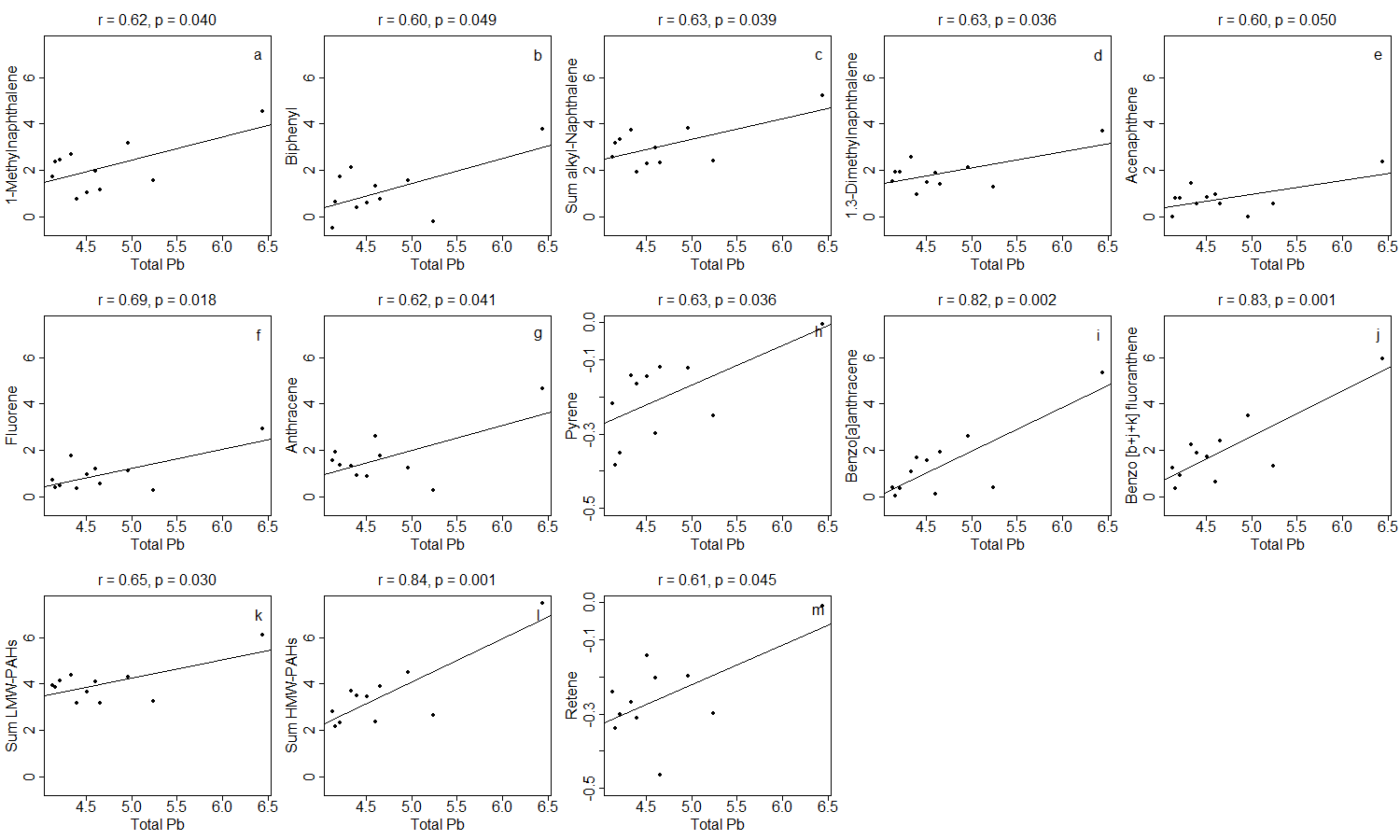


**Fig. S5a** Relationships between concentrations of Pb [µg g^-1^] and PAHs [ng g^-1^].


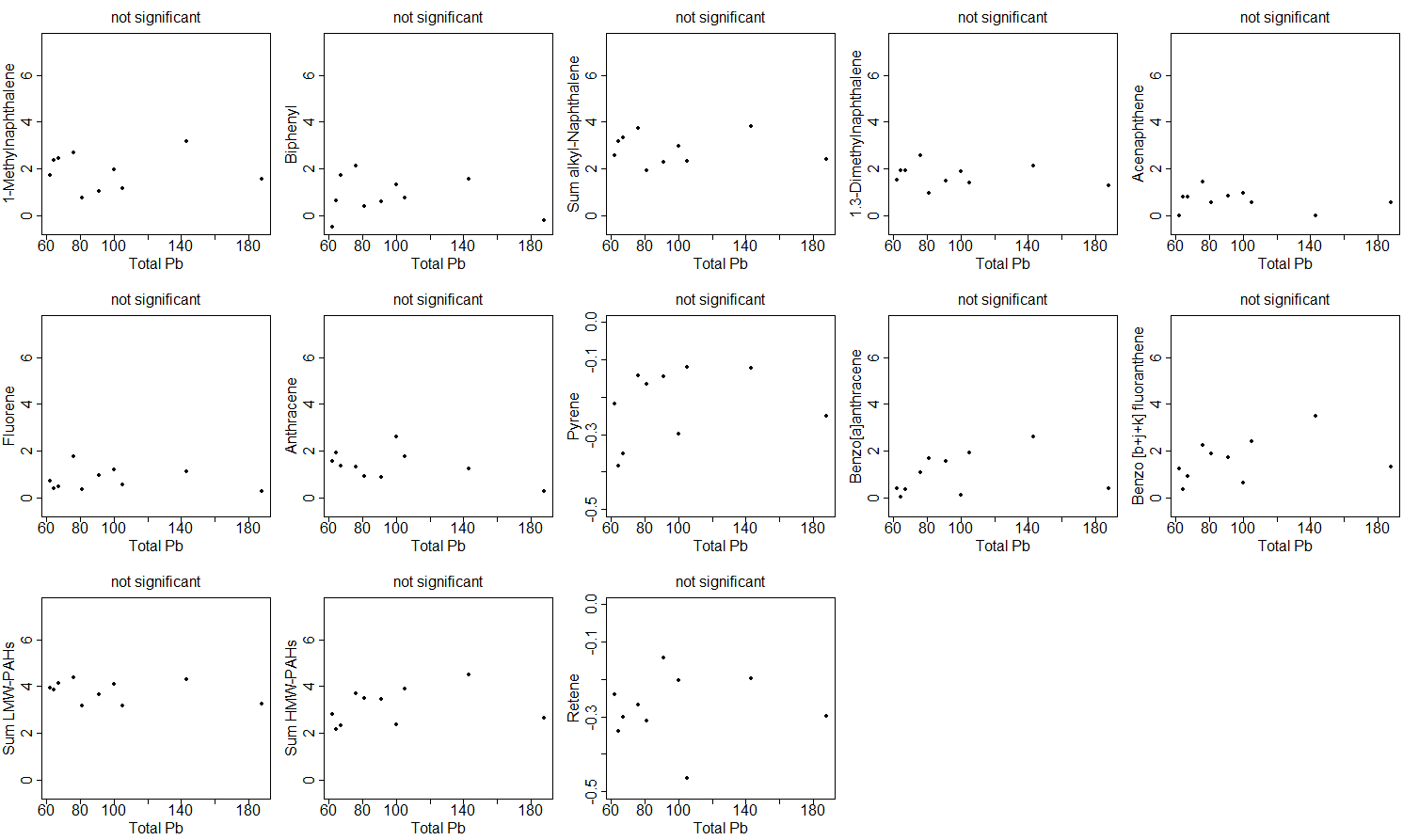


**Fig. S5b** Relationships between concentrations of Pb [µg g^-1^] and PAHs [ng g^-1^]. With outlier in Pb concentration removed.
